# Comparing endovascular techniques for left subclavian artery revascularization during zone 2 thoracic endovascular aortic repair for type B acute aortic syndromes: a retrospective cohort study

**DOI:** 10.3389/fcvm.2025.1566798

**Published:** 2025-06-04

**Authors:** Zizhong Zhang, Guangkun Sun, Jiacheng Ye, Bin Liu, Yongzheng Wang, Yuliang Li, Haiyang Chang

**Affiliations:** ^1^ Department of General Surgery, Wucheng People’s Hospital, Dezhou, China; ^2^ Imaging Department, Liaocheng Traditional Chinese Medicine Hospital, Liaocheng, China; ^3^ Department of Intervention Medicine, The First Hospital of Nanping Affiliated to Fujian Medical University, Nanping, Fujian, China; ^4^ Department of Intervention Medicine, The Second Hospital of Shandong University, Jinan, China; ^5^ Interventional Oncology Institute of Shandong University, Jinan, China

**Keywords:** thoracic endovascular aortic repair, left subclavian artery revascularization, Castor-branched stentgraft, chimney technique, fenestration technique

## Abstract

**Background:**

Castor-branched stent-graft, chimney stent, and fenestration techniques have been employed for left subclavian artery (LSA) revascularization during zone 2 thoracic endovascular aortic repair (TEVAR), but not recommended routinely. This study aimed to compare the safety and efficacy of these techniques.

**Methods:**

From February 2017 to June 2020, 133 patients with type B acute aortic syndromes undergoing LSA revascularization during zone 2 TEVAR were retrospectively enrolled. The primary outcomes include technical success, mortality, stroke and spinal cord ischemia. The secondary outcomes include aortic remodeling, LSA patency, freedom from aorta-related reintervention.

**Results:**

Fifty patients were treated with the Castor-branched stent-graft (Group A), 42 with the chimney technique (Group B), and 41 with the fenestration technique (Group C). The groups demonstrated a similar technical success rate, with 96% in Group A, 97.62% in Group B, and 95.12% in Group C. Despite a more predominant bird-beak configuration in group A (*p* = 0.003), no significant difference was observed in perioperative complications. Two TEVAR-related deaths occurred in Groups B and C, while none was reported in Group A. During the first two years of follow-up, favorable aortic remodeling was confirmed in all groups. Each group exhibited two cases of LSA occlusions. Aorta-related reintervention and mortality did not significantly differ among the groups.

**Conclusion:**

Endovascular techniques are feasible for LSA revascularization during zone 2 TEVAR, with favorable aortic remodeling. However, the durability of these procedures requires careful evaluation, given the potential concern of LSA occlusion.

## Introduction

Thoracic endovascular aortic repair (TEVAR) has been the primary therapeutic option for descending aorta pathologies since its initial report by Dake in 1994 (1). Currently, the application of TEVAR has expanded beyond the descending thoracic aorta to aortic arch pathologies. For patients with an inadequate proximal landing zone, zone 2 TEVAR with intentional coverage of the left subclavian artery (LSA) to extend the proximal landing zone is usually performed. Because of the increased risk of the upper extremities and spinal cord ischemia, and stroke events, current guidelines recommend routine LSA revascularization during zone 2 TEVAR (2–5).

TEVAR using carotid-subclavian bypass or subclavian transposition for LSA revascularization is considered to be the gold standard, with low morbidity and excellent long-term patency (6).

Several endovascular techniques have demonstrated safety and efficacy for LSA revascularization during zone 2 TEVAR (7–9). However, owing to the absence of long-term follow-up data, endovascular techniques for LSA revascularization during zone 2 TEVAR are not routinely recommended. This study aimed to evaluate the safety and efficacy of isolated LSA revascularization during zone 2 TEVAR, using a Castor-branched stent-graft, chimney stent, and the fenestration technique.

## Materials and methods

### Patients enrollment

The study was approved by our institutional ethics committee [March 1 2021, KYLL-2021(KJ)P-0140]. Given the retrospective design of the study, written informed consent was not required. The STROBE checklist was followed for the present study. From February 2017–June 2020, 133 patients diagnosed with acute complicated and high-risk type B aortic syndromes, including aortic dissection, intramural hematoma, and penetrating aortic ulcer, who underwent LSA revascularization during zone 2 TEVAR were retrospectively enrolled. The castor-branched stent-graft was indicated for patients with retrograde intramural hematoma or dissection involving the LSA orifice which was approved by China Food and Drug Administration. The fenestration technique was performed for the entry tears located near to the origin of the LSA, or too short intervals between the LSA and left common carotid artery contributed to an increased risk of endoleak. The chimney technique was offered to patients with ruptured dissection due to the requirement of urgent procedure limited employing the single branched stent-graft and performing fenestration. Unless otherwise specified, all techniques were offered without preference. Treatment decisions were made in a multi professional context, in accordance with characteristics of aortic pathology and patient preference. The rationale of different techniques is listed in Table 1. Castor-branched stent-grafts were deployed in 50 patients (Group A), the chimney technique was used in 42 patients (Group B), and fenestration technique was performed in 41 patients (Group C).

**Table 1:** Rationale for different endovascular techniques for LSA revascularization.

Rationale and characteristics	Castor-branched stent-graft	Chimney techniques	Fenestration techniques
Rationale	A single-branched stent-graft without distal or proximal bare stents	Parallel stent technology using commercial stents	In situ fenestration or pre-fenestration created using commercial stent-grafts
Advantages	Commercially available	No requirement of pre-customized, and being commonly used in emergency procedure	Simple handing, and no requirement of bared or covered stents
Disadvantages	The manufacturing time precludes their use in urgent cases	Increasing a risk of type Ia endoleak	Affecting the integrity of the stent-grafts

### Indications, outcomes and the definition

The primary outcomes include technical success, mortality, stroke and spinal cord ischemia rates. The secondary outcomes include aortic remodeling, LSA patency, freedom from aorta-related reintervention. TEVAR was indicated for type B acute aortic syndromes with rupture and/or branch vessel malperfusion, refractory pain and hypertension, and with high-risk radiographic and clinic feathers (aortic diameter >40 mm, false lumen diameter >20–22 mm, entry tear >10 mm and located at lesser curve, hemorrhagic pleural effusion, need for readmission, and reappearance of pain/symptoms) (3–5). The technical success was defined as complete excluding the entry tear with absence of complications (10). LSA patency was defined as diameter reduction <70% on follow up computed tomography angiography (CTA) (11). Aorta-related mortality was defined as all death due to the treated pathology and all deaths occurring ≤30 days of the procedure. Aorta-related reintervention was defined as a requirement of an additional surgical procedure due to the treated pathology (10).

### Endovascular procedure

All procedures were performed under general anesthesia in a hybrid operating room within 14 days after onset of symptoms by two operators with more than 20 years of experience in TEVAR. The mean interval from symptom onset to TEVAR procedure is approximately 2 days.

### Castor branched stent-graft

The left brachial artery (LBA) was cannulated using a 6F sheath for deployment of the branched section, while one side of the common femoral artery (CFA) was cannulated with a 5F sheath for aortic angiography percutaneously. The other side of the CFA was exposed, and a 5F MPA catheter was advanced into the exposed femoral artery, along with a guidewire through the sheath deployed in the LBA. Subsequently, a super-stiff guidewire was advanced into the ascending aorta via the exposed CFA. The stent-graft was then advanced into the descending aorta, and simultaneously, the traction wire of the branched section was pulled out from the tail of the 5F MPA catheter. The stent-graft was delivered into the aortic arch, with the outer sheath left in the descending aorta. The soft sheath was removed, and the branching section was drawn into the LSA. The stent-graft trunk was released by withdrawing the trigger guidewire, and the branched section was deployed by removing the traction wire.

### Chimney technique

The LBA was cannulated with a 6F sheath percutaneously, followed by inserting a 5F pigtail catheter into the ascending aorta for aortic angiography. Surgical cut down of the CFA was performed. The Ankura stent-graft (Life-tech Scientific, Shenzhen, China), with a proximal bare stent ensuring a better anchor, was delivered to the planned location in the aortic arch along with a super-stiff guidewire deployed in the exposed CFA, and then zone 2 deployment was performed. A stiff guidewire was simultaneously inserted into the ascending aorta via the 5F pigtail catheter. Subsequently, a chimney stent, one kind of bare self-expanding metal stent, was advanced into the LSA along with the stiff guidewire. The chimney stent was deployed at the planned location with a 10 mm protrusion into the aortic arch.

### Fenestration technique

A 5F pigtail catheter was advanced into the ascending aorta for angiography through a 6F sheath deployed in the LBA percutaneously. The centerline length between the top of the proximal landing zone and the origin of the LSA was analyzed to determine the center of the fenestration. The proximal segment of the Ankura stent-graft was unsheathed on the back table in sterile conditions. The fenestration, sized comparably to ostium of the LSA, was premarked in the stent-graft, and then created in line with the “8”-shaped radio-opaque marker. The stent-graft was reloaded in the sheath with the fenestration on the outer side by tightening the sutures around the stent graft to avoid distortion. Clock position was used to determine the LSA position on the Volume-rendering image, and minor rotations was performed during the release of the first segment to ensure well alignment of the fenestration to the origin of the LSA.

Aortic angiography was performed to confirm the exclusion of the dissection and patency of the aortic arch branches for patients among these groups.

### Follow-up

Follow-up was repeated after 1, 3, 6, and 12 months, and then annually. In the absence of contraindications, CTA was the preferred imaging modality for follow-up evaluations.

### Statistical analysis

Continuous variables were recorded as mean ± standard deviation and analyzed by ANOVA. Categorical variables, expressed as numbers and proportions, were compared using Pearson's chi-square test, Fisher's exact test, or the Wilcoxon rank-sum test. Kaplan–Meier curves were used to determine the possibilities of events such as LSA occlusion, aortic-related reintervention, and mortality. A *p*-value of <0.05 was considered statistically significant. Statistical analyses were conducted using SPSS Statistics 19.0 (IBM Corp, Armonk, NY, USA).

## Results

### Baseline characteristics

The mean age of patients was 56 ± 11 years, 54 ± 14 years, and 55 ± 11 years in group A, B, and C. The majority of patients in all groups were male and had a history of hypertension. Groups A, B, and C had 39, 34, and 33 patients who were smokers. Trauma caused one dissection in group A, and one patient underwent open surgery for an ascending aortic aneurysm before TEVAR in group B. The clinical and radiographic features showed no significant differences among the groups. Further details are presented in Table 2.

**Table 2:** Baseline characteristics of patients.

Baseline characteristics	Castor, *n* = 50	Chimney, *n* = 42	Fenestration, *n* = 41	*p*
**Age, y**	56 ± 11	54 ± 14	55 ± 11	0.585
**Gender, m**	41	31	29	0.418
Co-morbidity, *n*				
Hypertension	40	36	35	0.733
CAD	2	2	1	1
DM	2	2	0	0.551
Hyperlipidemia	21	17	18	0.975
Chronic obstructive pulmonary disease	4	3	5	0.747
Current or former smoker	39	34	33	0.928
Others	4	4	3	1
Aortic pathologies, *n*				
Aortic dissection	**40**	**32**	**31**	0.869
Penetrating aortic ulcer	5	3	4	0.866
Intramural hematoma	5	7	6	0.629
LSA involvement	13	15	11	0.552
**Rupture/malperfusion**	**0**	**5**	**0**	
**Refractory pain/hypertension**	**33**	**25**	**27**	
**High-risk radiographic feature**	**17**	**12**	**14**	**0**.**053**

CAD, coronary artery disease; DM, diabetes mellitus; LSA, left subclavian artery.

The indications for TEVAR among the groups are in bold.

### Perioperative outcome

Perioperative outcomes are detailed in Table 3. Technical success was achieved in 48 (96%), 41 (97.62%), and 39 (95.12%) patients in Groups A, B, and C. Procedure and fluoroscopy times showed no significant differences among the groups. Patients in Group C experienced lower blood loss than those in the other two groups. Group B utilized more contrast loads than the other two groups, with a negligible difference. Bird-beak configuration (BBC) was more predominant in Group A. TEVAR-related death and length of hospital stay were comparable among these groups. In group B, one sudden death occurred three days after TEVAR, and the other patient died within 30 days after TEVAR. In group C, both patients died within one week after TEVAR. Perioperative complications, including immediate endoleaks, spinal cord ischemia, and stroke, did not differ statistically among the groups. In Group A, two type I endoleaks and one distal stent-induced new entry were observed before discharge. One type I endoleak and two type II endoleaks were confirmed in Group B, and two type I endoleaks were found in group C.

**Table 3:** Peri-operative outcome.

Peri-operative outcomes	Castor, *n* = 50	Chimney, *n* = 42	Fenestration, *n* = 41	*p*
Procedure time, minutes	122.60 ± 28.23	117.38 ± 31.38	115.36 ± 28.64	0.476
Fluoroscopy time, minutes	21.04 ± 5.39	20.57 ± 4.34	20.12 ± 3.79	0.640
Blood loss, ml	35.1 ± 7.32	31.54 ± 8.94	25.73 ± 8.56	<0.001
Contrast load, ml	104 ± 8.02	108.45 ± 12.27	104.02 ± 9.5	0.062
BBC, *n*	28	10	12	0.003
Hospital length of stay, *d*	17.98 ± 8.63	16.38 ± 6.02	15.29 ± 3.12	0.144
Primary outcomes				
Technical success, *n* (%)	49 (98)	41 (97.62)	39 (95.12)	0.689
In-hospital mortality, *n*	0	2	2	0.225
Aorta-related mortality, *n*	1	2	2	0.97
Spinal cord ischemia	0	0	0	N/A
Stroke	1	0	0	1
Secondary outcomes				
LSA patency, %	96	95.2	95.1	0.965
Aorta-related reintervention, *n*	2	2	2	0.965
Immediate endoleak	2	3	2	0.889
Other complications	3	3	3	1
Parameters of stent-graft				
No. of endografts placed				
One	47	39	41	0.284
Two	3	3	0	
Oversize, %	7.84 ± 2.7	5.06 ± 1.88	5.16 ± 1.84	<0.001
Length of coverage, mm	203.2 ± 13.62	201.67 ± 18.73	195.61 ± 8.38	0.034

### Aortic remodeling

**Figure 1:**
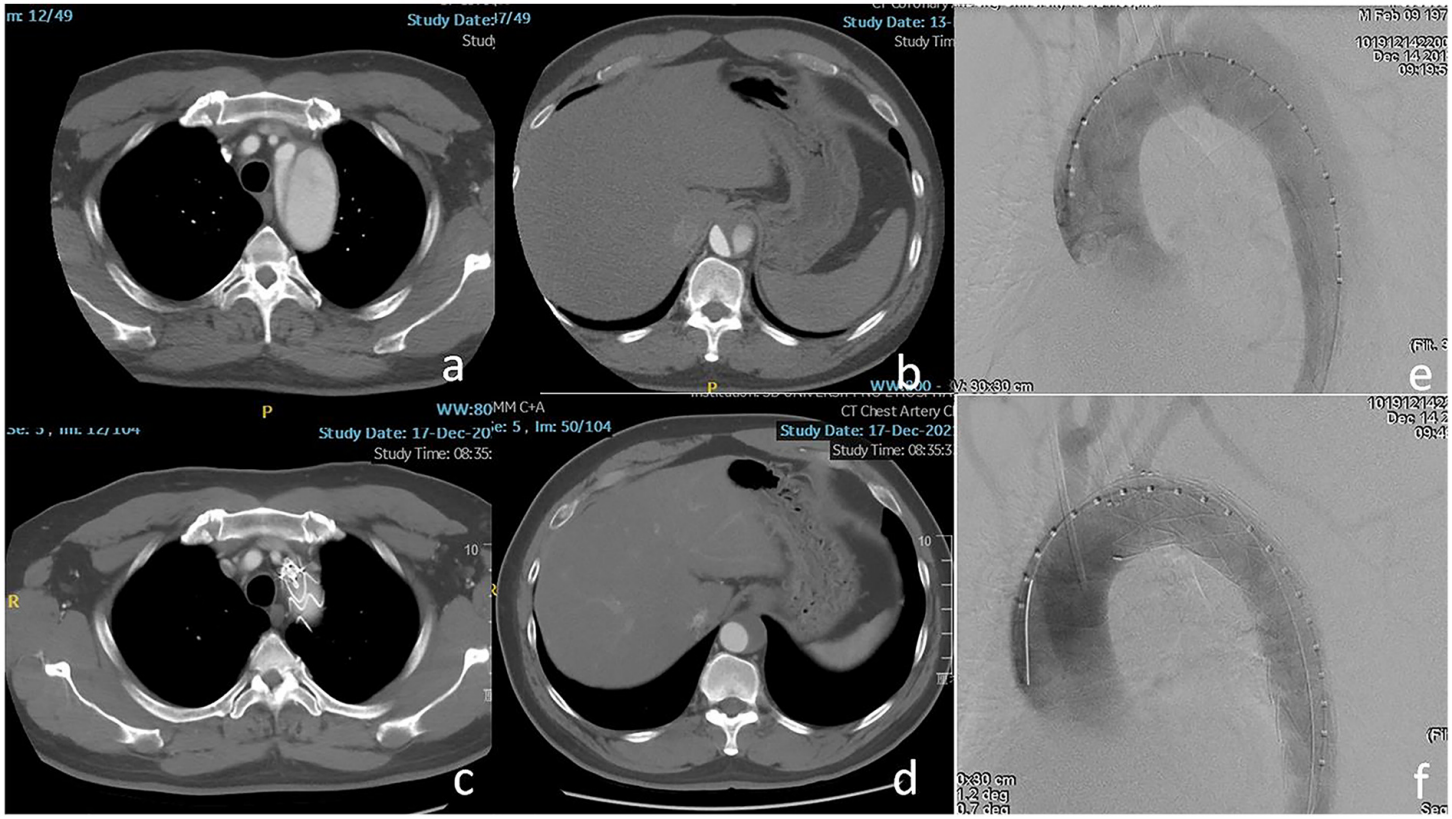
**(a,b)** The dissection involved the ostium of the left subclavian artery (LSA) and the visceral segment of the aorta. **(c,d)** The LSA was patent and complete thrombosis of the false lumen was confirmed at the level of the visceral segment of the aorta after 2 years. **(e,f)** The entry tear was excluded completely using Castor-branched stent-graft.

**Figure 2:**
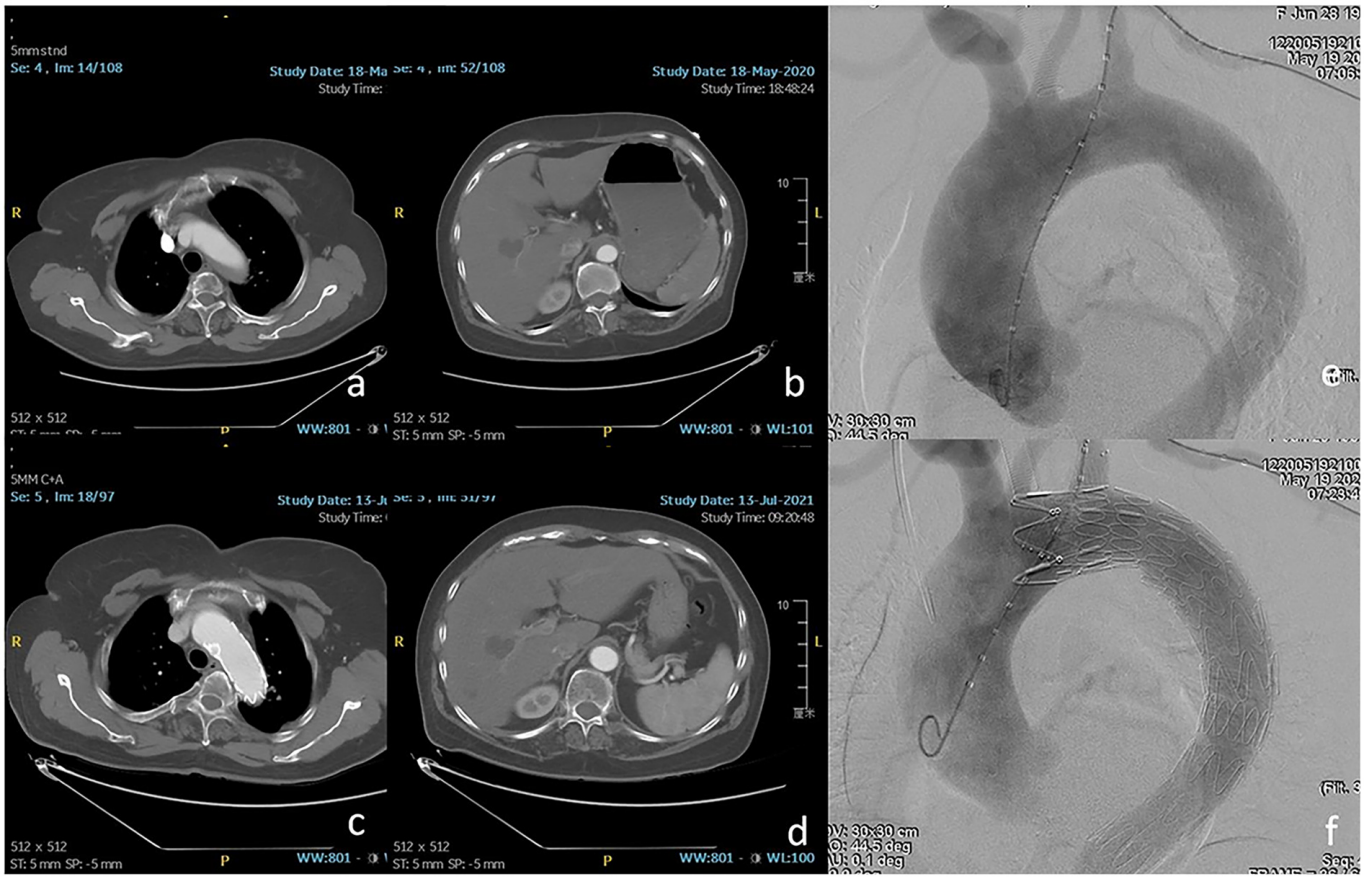
**(a,b)** The orifice of the LSA was involved by intramural hematoma. **(c,d)** One year after thoracic endovascular aortic repair (TEVAR), favorable aortic remodeling and patent LSA were detected. **(e,f)** Zone 2 landing was performed using chimney stent to preserve the LSA.

**Figure 3:**
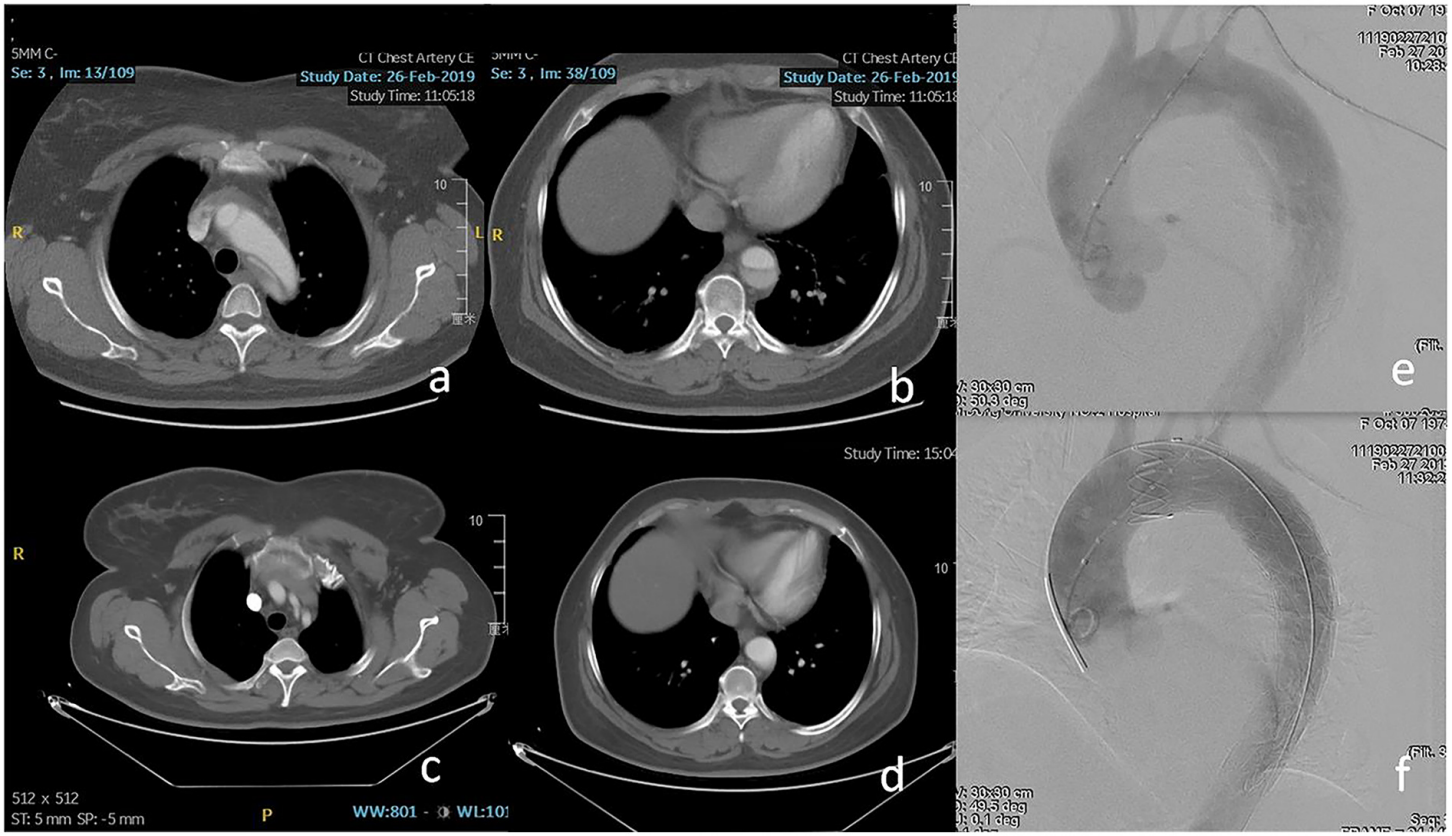
**(a,b)** Retrograde dissection involved the inner curve of zone 2 and the visceral segment of the aorta. **(c,d)** Eight months after TEVAR, the patency of the LSA and complete thrombosis of the segment distal to the stent-graft was found on computed tomography angiography (CTA). **(e,f)** The fenestrated stent-graft was deployed in zone 2 to exclude the entry tear and preserve the LSA.

Thrombosis of the false lumen is detailed in Table 4. During the first two years of follow-up, complete thrombosis of the false lumen in the stented segment of the thoracic aorta was confirmed in all patients. Partial and complete thrombosis of the false lumen increased significantly in both the aorta distal to the stent-graft and the visceral segment of the aorta. Thrombosis of the false lumen is illustrated in Figures 1–3. The mean change in the maximum transaortic diameter of the aorta distal to the stent-graft was −1.72 mm in Group A, −2.05 mm in Group B, and −1.83 mm in Group C. Regarding the level of the visceral segment of the aorta, the mean change was 1.2 mm in Group A, 1.1 mm in Group B, and 1 mm in Group C. The aorta was stable and shrinking in the majority of patients in the aorta distal to the stent-graft (92.5% in group A, 93.75% in group B, and 93.5% in group C) and the visceral segment of the aorta (89.3% in group A, 91.3% in group B, and 91.7% in group C). No significant difference was detected among the groups during follow up. Further details are presented in Table 5.

**Table 4:** Thrombosis of the false lumen of the aorta during two years of follow-up.

False lumen thrombosis of the aorta	Castor		Chimney		Fenestration	
The aorta distal to the stent graft	Pre-TEVAR	Post-TEVAR	Pre-TEVAR	Post-TEVAR	Pre-TEVAR	Post-TEVAR
Complete, *n* (%)	0 (0)	18 (45)	0 (0)	10 (31.3)	0 (0)	12 (38.7)
Partial, *n* (%)	5 (12.5)	16 (40)	4 (12.5)	9 (28.1)	5 (16.1)	11 (35.5)
Patent, *n* (%)	35 (87.5)	6 (15)	28 (87.5)	13 (40.6)	26 (83.9)	8 (25.8)
*P*	<0.001		<0.001		<0.001	
The visceral segment of the aorta	Pre-TEVAR	Post-TEVAR	Pre-TEVAR	Post-TEVAR	Pre-TEVAR	Post-TEVAR
Complete, *n* (%)	0 (0)	7 (25)	0 (0)	5 (21.7)	0 (0)	6 (25)
Partial, *n* (%)	3 (10.7)	12 (42.9)	3 (13)	8 (34.8)	2 (8.3)	10 (41.7)
Patent, *n* (%)	25 (89.3)	9 (32.1)	20 (87)	10 (43.5)	22 (91.7)	8 (33.3)
*P*	<0.001		0.001		<0.001	

**Table 5:** Aortic remodeling during two years of follow-up.

Aortic remodeling	Castor	Chimney	Fenestration	*p*
Transaortic diameter change in the aorta distal to the stent graft, mm	−1.72 ± 3.24	−2.05 ± 2.81	−1.83 ± 1.46	0.923
Shrinkage, % (*N*)	30 (12/40)	31.25 (10/32)	29 (9/31)	
Stable, % (*N*)	62.5 (25/40)	62.5 (20/32)	64.5 (20/31)	
Growth, % (*N*)	7.5 (3/40)	6.25 (2/32)	6.5 (2/31)	0.992
Expansion of the true lumen, mm	3.56 ± 0.81	3.94 ± 0.67	3.94 ± 0.77	0.067
Shrinkage of the false lumen, mm	5.28 ± 0.96	5.99 ± 0.78	5.77 ± 0.91	0.87
Transaortic diameter change in the visceral segment of the aorta, mm	1.2 ± 1.63	1.1 ± 1.79	1.00 ± 1.24	0.834
Shrinkage, % (*N*)	35.7 (10/28)	34.8 (8/23)	29.2 (7/24)	
Stable, % (*N*)	53.6 (15/28)	56.5 (13/23)	62.5 (15/24)	
Growth, % (*N*)	10.7 (3/28)	8.7 (2/23)	8.3 (2/24)	0.984
Expansion of the true lumen, mm	2.61 ± 0.74	2.74 ± 0.45	2.92 ± 0.65	0.221
Shrinkage of the false lumen, mm	1.41 ± 0.74	1.64 ± 0.34	1.92 ± 0.55	0.538

### Follow-up outcome

**Figure 4:**
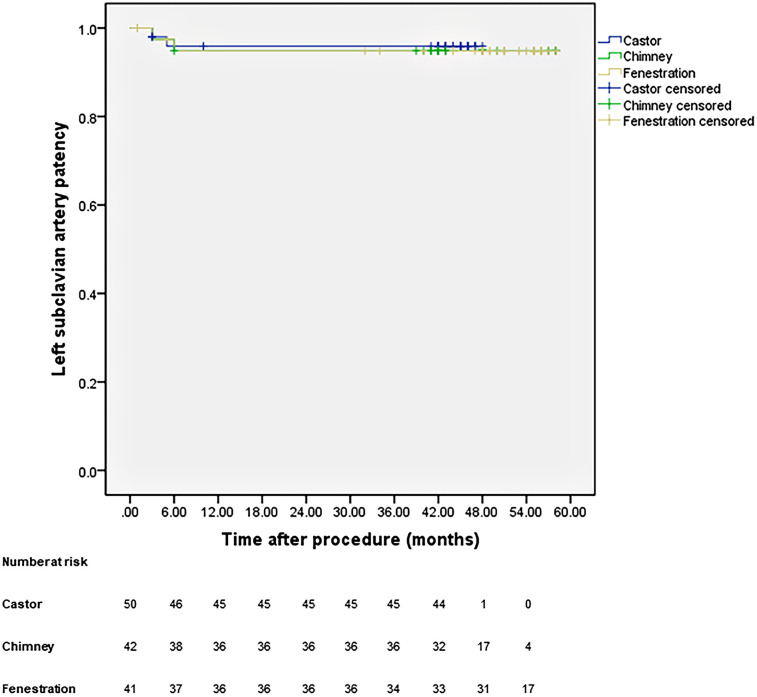
Cumulative Kaplan–Meier estimates of the LSA patency during midterm follow-up.

**Figure 5:**
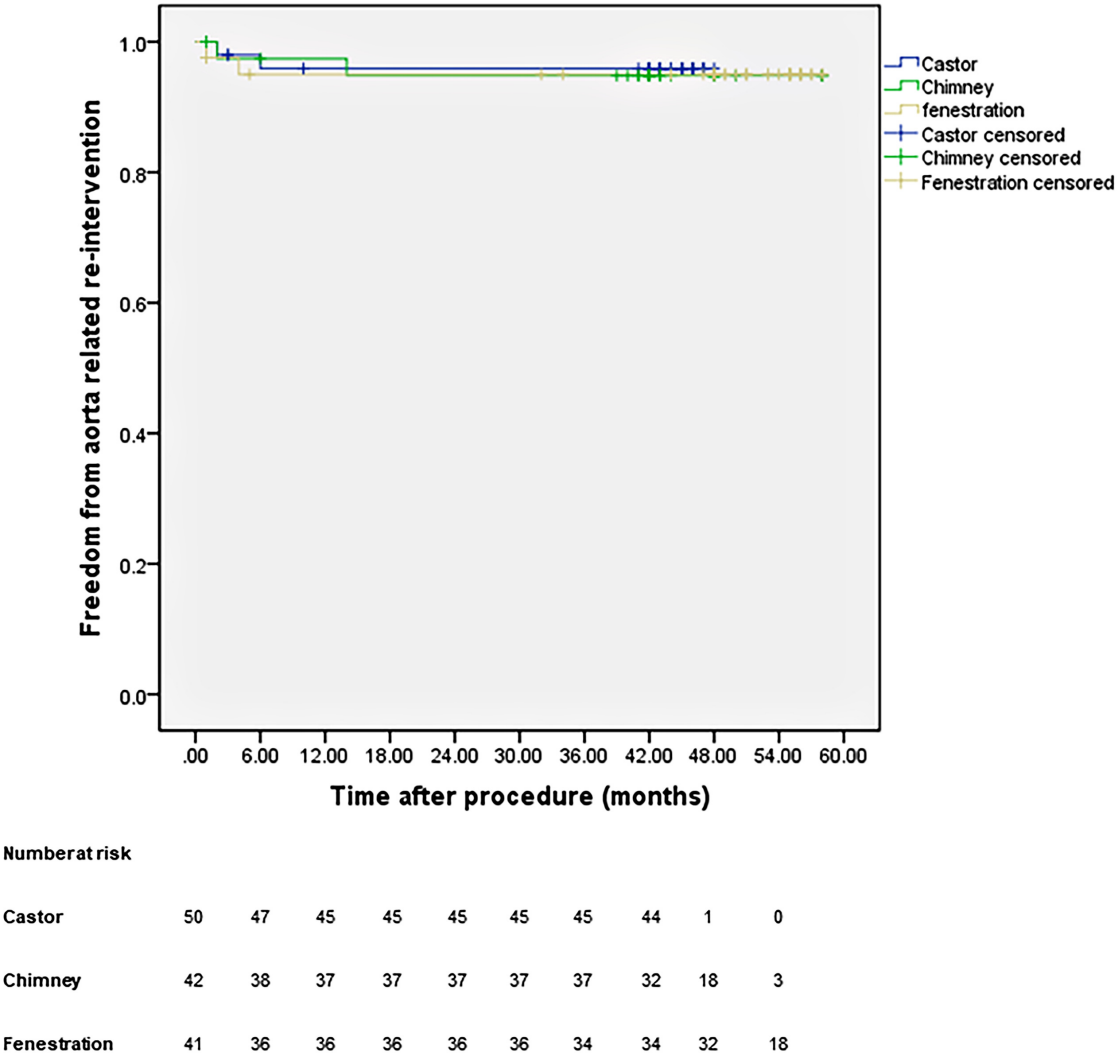
Cumulative Kaplan–Meier estimates of the aorta-related reintervention during midterm follow-up.

**Figure 6:**
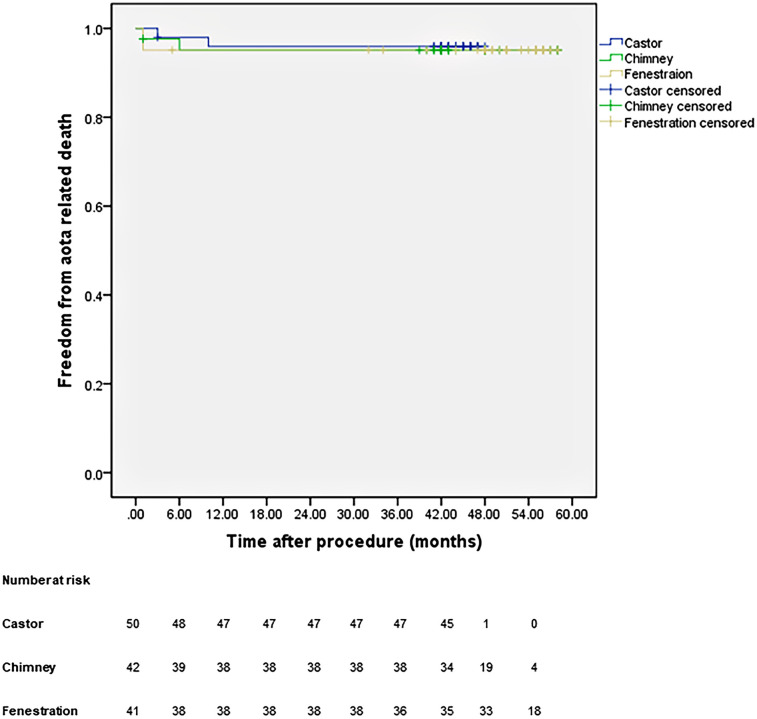
Cumulative Kaplan–Meier estimates of the aorta-related death during midterm follow-up.

All patients were closely followed according to the protocol. Figure 4 shows the LSA patency for all patients with no significant difference in the three groups (*p* *=* 0.965). The LSA occlusion without symptoms was detected in two patients in each group. Figure 5 provides details on aorta-related reinterventions during follow-up, with no statistical significance observed among the groups (*p* *=* 0.965). In Groups A and C, one retrograde type A dissection was found, leading to an open repair in both cases. Two additional stent-grafts were deployed in Group B because of persistent endoleaks 14 months after TEVAR. Aorta-related deaths due to Aortic rupture and/or retrograde dissection leading to fatal cardiac tamponade occurred in the following patients: one in Group A, two in Group B, and two in Group C. The details are illustrated in Figure 6. No significant difference was observed among the groups during follow-up (*p* *=* 0.97).

## Discussion

During zone 2 TEVAR, intentional coverage of the LSA increased the risk of upper extremity and spinal cord ischemia, and stroke (12–14). Therefore, several endovascular techniques, including single-branched stent-graft, chimney stent, and fenestration techniques, are used for LSA revascularization. Despite carotid-subclavian bypass or transposition being the gold standard for LSA revascularization during zone 2 TEVAR (2, 15), our study utilized different endovascular techniques to preserve the LSA during zone 2 TEVAR. The Castor-branched stent-graft, chimney stent, and fenestration techniques are safe, effective, and minimally invasive procedures with a low incidence of complications and a high technical success rate. All techniques facilitated favorable aortic remodeling during the first two years of follow-up, although the Castor-branched stent-graft exhibited a higher incidence of BBC.

A Castor-branched stent-graft is a single-branched stent-graft without distal or proximal bare stents. It has received approval for the simultaneous exclusion of the entry tears and LSA preservation during zone 2 TEVAR. Jing et al. reported the safety and effectiveness of Castor-branched stent-grafts for deployment proximal to the LSA orifice (7). Compared with the other two endovascular treatments, this type of stent-graft seems to be associated with a higher incidence of BBC, a factor considered to elevate the risk of type I endoleaks and stent-graft collapse or fracture (16–18). Designing a stent-graft without a proximal bare stent, withdrawing the traction wire to deploy the branch section, and using a greater oversizing may contribute to this dilemma (7, 16, 19). Despite the higher incidence of BBC in Castor-branched stent-grafts, no statistical difference in complications was observed among the groups. The relatively high incidence of BBC seems not to increase the risk of clinical events. Long-term follow-up is required to confirm these findings.

Technical success was achieved in 41 patients who received the chimney stent, with three endoleaks detected immediately after TEVAR. However, no reintervention was required for these patients as they remained asymptomatic during follow-up. Compared to previous reports, our center observed a slightly lower incidence of endoleaks in patients treated with chimney stents (20, 21). The diminished gutter between the bare chimney stent and the main aortic stent-graft seems to contribute to a reduced risk of endoleaks. However, achieving complete gutter sealing with current techniques remains challenging (20, 22, 23). Despite concerns that a bare metal chimney stent might increase the incidence of LSA occlusion (22, 24), no significant difference was observed among the groups during follow-up. Notably, selection criteria for chimney stents have not been established.

For patients requiring intentional coverage of the LSA to extend the proximal landing zone during zone 2 TEVAR, both on-the-table and *in situ* fenestration techniques have demonstrated durability and safety for isolated LSA revascularization during midterm follow-up (9, 14, 25, 26). Comparable to previous studies, technical success was achieved in 39 patients, with two type Ia endoleaks detected immediately after the procedure. One persistent type Ia endoleak was repaired using a double fenestration technique to extend the proximal landing to zone 1. The other type Ia endoleak disappeared spontaneously on CTA before discharge, suggesting that thrombosis and aortic remodeling occur over time (27). A “Wait and Watch” strategy could be considered for slight immediate type Ia endoleak.

Previous studies suggest that complete endovascular techniques for LSA revascularization during zone 2 TEVAR achieved outcomes comparable to those of carotid-subclavian bypass or transposition (6, 28, 29). For branched stent-graft and chimney technique, ensuring alignment and fully expanding of the branched section or the chimney stent is challenging during zone 2 TEVAR. For fenestration technique, aligning the fenestration with the origin of the LSA is difficult, and unexpected covering the origin of the LSA is not rare. Therefore, endovascular techniques pose an increased risk of clinically silent LSA occlusion. While single-branched stent-grafts have been approved for managing aortic arch pathologies, the manufacturing time precludes their use in urgent cases (7). The chimney technique, using a bare stent, appears to be associated with an increased risk of endoleak for entry tears at the outer curve. The fenestration technique affects the integrity of the stent-graft, emphasizing the need to evaluate the applicability and durability of fenestrated stent-grafts through additional data during long-term follow-ups. Individualized adjustments are crucial when preserving the LSA during zone 2 TEVAR to account for diverse conditions.

The present study has a few limitations. First, being a single-center study with a limited number of patients, the selection basis should be considered when interpreting the outcomes. Second, the absence of a comparison between endovascular techniques and carotid-subclavian bypass surgery calls for further randomized control trials to evaluate the applicability and durability of complete endovascular techniques for LSA revascularization during zone 2 TEVAR. Third, the chimney technique was performed using a self-expanded bare metal stent, and the establishment of selection criteria for the chimney stent requires more data during long-term follow-up.

Endovascular techniques, including single-branched stent-graft, chimney, and fenestration techniques, are feasible for LSA revascularization during zone 2 TEVAR, with favorable aortic remodeling during midterm follow-up. It is necessary to evaluate whether endovascular techniques could offer outcomes comparable to carotid to subclavian artery bypass during zone 2 TEVAR for type B acute aortic syndromes, given the potential concern of LSA occlusion.

## Data Availability

The raw data supporting the conclusions of this article will be made available by the authors, without undue reservation.

## References

[1] Dake MD Miller DC Semba CP Mitchell RS Walker PJ Liddell RP. Transluminal placement of endovascular stent-grafts for the treatment of descending thoracic aortic aneurysms. N Engl J Med. (1994) 331(26):1729–34. 10.1056/NEJM199412293312601 7984192

[2] Matsumura JS Lee WA Mitchell RS Farber MA Murad MH Lumsden AB The society for vascular surgery practice guidelines: management of the left subclavian artery with thoracic endovascular aortic repair. J Vasc Surg. (2009) 50(5):1155–58. 10.1016/j.jvs.2009.08.090 19878791

[3] Lombardi JV Hughes GC Appoo JJ Bavaria JE Beck AW Cambria RP Society for vascular surgery (SVS) and society of thoracic surgeons (STS) reporting standards for type B aortic dissections. J Vasc Surg. (2020) 71(3):723–47. 10.1016/j.jvs.2019.11.013 32001058

[4] Mazzolai L Teixido-Tura G Lanzi S Boc V Bossone E Brodmann M 2024 ESC guidelines for the management of peripheral arterial and aortic diseases. Eur Heart J. (2024) 45(36):3538–700. 10.1093/eurheartj/ehae179 39210722

[5] Czerny M Grabenwöger M Berger T Aboyans V Della Corte A Chen EP EACTS/STS guidelines for diagnosing and treating acute and chronic syndromes of the aortic organ. Ann Thorac Surg. (2024) 118(1):5–115. 10.1016/j.athoracsur.2024.01.021 38416090

[6] Mandigers TJ de Beaufort HWL Smeenk HG Vos JA Heijmen RH. Long-term patency of surgical left subclavian artery revascularization. J Vasc Surg. (2022) 75:1977–1984.e1. 10.1016/j.jvs.2021.12.078 35090990

[7] Jing Z Lu Q Feng J Zhou J Feng R Zhao Z Endovascular repair of aortic dissection involving the left subclavian artery by castor stent graft: a multicentre prospective trial. J Vasc Surg. (2021) 73(1):345. 10.1016/j.jvs.2020.11.013 33183920

[8] Ramdon A Patel R Hnath J Yeh CC Darling RC. Chimney stent graft for left subclavian artery preservation during thoracic endograft placement. J Vasc Surg. (2020) 71(3):758–66. 10.1016/j.jvs.2019.05.049 32089209

[9] Kuo HS Huang JH Chen JS. Handmade stent graft fenestration to preserve left subclavian artery in thoracic endovascular aortic repair. Eur J Cardiothorac Surg. (2019) 56(3):587–94. 10.1093/ejcts/ezz049 30809647

[10] Fillinger MF Greenberg RK McKinsey JF Chaikof EL. Reporting standards for thoracic endovascular aortic repair (TEVAR). J Vasc Surg. (2010) 52(4):1022–33. 10.1016/j.jvs.2010.07.008 20888533

[11] Che WQ Dong H Jiang XJ Peng M Zou YB Qian HY Stenting for left subclavian artery stenosis in patients scheduled for left internal mammary artery-coronary artery bypass grafting. Catheter Cardiovasc Interv. (2016) 87(Suppl 1):579–88. 10.1002/ccd.26477 26914391

[12] Waterford SD Chou D Bombien R Uzun I Shah A Khoynezhad A. Left subclavian arterial coverage and stroke during thoracic aortic endografting: a systematic review. Ann Thorac Surg. (2016) 101(1):381–89. 10.1016/j.athoracsur.2015.05.138 26588864

[13] Xie W Xue Y Li S Jin M Zhou Q Wang D. Left subclavian artery revascularization in thoracic endovascular aortic repair: single center’s clinical experiences from 171 patients. J Cardiothorac Surg. (2021) 16(1):207. 10.1186/s13019-021-01593-w 34330305 PMC8325210

[14] Canaud L Morishita K Gandet T Sfeir J Bommart S Alric P Homemade fenestrated stent-graft for thoracic endovascular aortic repair of zone 2 aortic lesions. J Thorac Cardiovasc Surg. (2018) 155(2):488–93. 10.1016/j.jtcvs.2017.07.045 28867380

[15] Voigt SL Bishawi M Ranney D Yerokun B McCann RL Hughes GC. Outcomes of carotid-subclavian bypass performed in the setting of thoracic endovascular aortic repair. J Vasc Surg. (2019) 69(3):701–09. 10.1016/j.jvs.2018.07.022 30528402

[16] Boufi M Alexandru G. Commentary: bird beak after TEVAR: hostile or benign sign? J Endovasc Ther. (2019) 26(6):779–81. 10.1177/1526602819881138 31736423

[17] Ueda T Fleischmann D Dake MD Rubin GD Sze DY. Incomplete endograft apposition to the aortic arch: bird-beak configuration increases risk of endoleak formation after thoracic endovascular aortic repair. Radiology. (2010) 255(2):645–52. 10.1148/radiol.10091468 20413775 PMC6939947

[18] Marrocco-Trischitta MM Spampinato B Mazzeo G Mazzaccaro D Milani V Alaidroos M Impact of the bird-beak configuration on postoperative outcome after thoracic endovascular aortic repair: a meta-analysis. J Endovasc Ther. (2019) 26(6):771–78. 10.1177/1526602819865906 31364458

[19] Banno H Akita N Fujii T Tsuruoka T Takahashi N Sugimoto M Proximal bare stent may reduce bird-beak configuration, which is associated with distal migration of stent graft in the aortic arch. Ann Vasc Surg. (2019) 56:108–13. 10.1016/j.avsg.2018.08.081 30342207

[20] Zhang L Wu MT Zhu GL Feng JX Song C Li HY Off-the-shelf devices for treatment of thoracic aortic diseases: midterm follow-up of TEVAR with chimneys or physician-made fenestrations. J Endovasc Ther. (2020) 27(1):132–42. 10.1177/1526602819890107 31789078

[21] Wang T Shu C Li QM Li M Li X He H First experience with the double chimney technique in the treatment of aortic arch diseases. J Vasc Surg. (2017) 66(4):1018–27. 10.1016/j.jvs.2017.02.035 28502544

[22] Lindblad B Bin Jabr A Holst JJ Malina M. Chimney grafts in aortic stent grafting: hazardous or useful technique? Systematic review of current data. Eur J Vasc Endovasc Surg. (2015) 50(6):722–31. 10.1016/j.ejvs.2015.07.038 26371416

[23] Ding H Liu Y Xie N Fan R Luo S Huang W Outcomes of chimney technique for preservation of the left subclavian artery in type B aortic dissection. Eur J Vasc Endovasc Surg. (2019) 57(3):374–81. 10.1016/j.ejvs.2018.09.005 30297205

[24] Bosiers MJ Donas KP Mangialardi N Torsello G Riambau V Criado FJ European multicenter registry for the performance of the chimney/snorkel technique in the treatment of aortic arch pathologic conditions. Ann Thorac Surg. (2016) 101(6):2224–30. 10.1016/j.athoracsur.2015.10.112 26794885

[25] Chassin-Trubert L Mandelli M Ozdemir BA Alric P Gandet T Canaud L. Midterm follow-up of fenestrated and scalloped physician-modified endovascular grafts for zone 2 TEVAR. J Endovasc Ther. (2020) 27(3):377–84. 10.1177/1526602819881128 31645219

[26] Sonesson B Dias N Abdulrasak M Resch T. Midterm results of laser generated *in situ* fenestration of the left subclavian artery during thoracic endovascular aneurysm repair. J Vasc Surg. (2019) 69(6):1664–69. 10.1016/j.jvs.2018.09.052 30591297

[27] Grabenwöger M Alfonso F Bachet J Bonser R Czerny M Eggebrecht H Thoracic endovascular aortic repair (TEVAR) for the treatment of aortic diseases: a position statement from the European association for cardio-thoracic surgery (EACTS) and the European society of cardiology (ESC), in collaboration with the European association of percutaneous cardiovascular interventions (EAPCI). Eur Heart J. (2012) 33(13):1558–63. 10.1093/eurheartj/ehs074 22561257

[28] D'Oria M Kärkkäinen JM Tenorio ER Oderich GS Mendes BC Shuja F Perioperative outcomes of carotid-subclavian bypass or transposition versus endovascular techniques for left subclavian artery revascularization during nontraumatic zone 2 thoracic endovascular aortic repair in the vascular quality initiative. Ann Vasc Surg. (2020) 69:17–26. 10.1016/j.avsg.2020.05.062 32505683

[29] Mandigers TJ Allievi S Jabbour G Gomez-Mayorga JL Caron E Giles KA Comparison of open and endovascular left subclavian artery revascularization for zone 2 thoracic endovascular aortic repair. J Vasc Surg. (2024) 80(5):1425–1436.e3. 10.1016/j.jvs.2024.06.018 38880180

